# Unfolding the Complexity of Exosome–Cellular Interactions on Tumour Immunity and Their Clinical Prospects in Nasopharyngeal Carcinoma

**DOI:** 10.3390/cancers16050919

**Published:** 2024-02-24

**Authors:** Paak-Ting Chak, Ngar-Woon Kam, Tsz-Ho Choi, Wei Dai, Dora Lai-Wan Kwong

**Affiliations:** 1 Department of Clinical Oncology, Centre of Cancer Medicine, School of Clinical Medicine, LKS Faculty of Medicine, The University of Hong Kong, Hong Kong 999077, China; z1rux@connect.hku.hk (P.-T.C.); yvonnekam@hksccb.hk (N.-W.K.); cth5544@connect.hku.hk (T.-H.C.); 2 Laboratory for Synthetic Chemistry and Chemical Biology Limited, Hong Kong Science Park, New Territories, Hong Kong 999077, China; 3 Clinical Oncology Center, The University of Hong Kong-Shenzhen Hospital, Shenzhen 518053, China

**Keywords:** nasopharyngeal carcinoma, tumour microenvironment, exosomes, extracellular vesicles, immune checkpoint inhibitors, cellular therapy, cancer vaccine, liquid biopsy, biomarkers

## Abstract

**Simple Summary:**

Nasopharyngeal carcinoma (NPC) is a malignancy prevalent in Southeast Asia with the highest metastatic rate amongst other head and neck cancers. The absence of symptoms and unique anatomical positioning often result in late diagnosis, with resistance to chemo/radiotherapy further impacting its prognosis. While immunotherapies such as immune-checkpoint inhibitors (ICIs) and cytotoxic-T-lymphocytes (CTLs)-based cellular therapy have been progressively developed and approved, the mechanisms underlying the immunosuppressive tumour microenvironment (TME) in NPC remain poorly understood. Exosomes, small extracellular vesicles ranging in size from 30 to 150 nm, have emerged as important mediators of cell–cell communications within the TME. Accumulating evidence points tumour-derived exosomes (TEX) to NPC immune evasion and progression. Additionally, the detectability of TEX-bound NPC-specific markers in bodily fluids shows promise in enabling early diagnosis and prognostication. In this review, we aim to uncover TEX-mediated cellular–pathogenic pathways to open new avenues for an immunotherapeutic regimen in NPC and highlight the potential of exosome-based biomarkers in bettering NPC outcomes.

**Abstract:**

Nasopharyngeal carcinoma (NPC) is an epithelial malignancy situated in the posterolateral nasopharynx. NPC poses grave concerns in Southeast Asia due to its late diagnosis. Together with resistance to standard treatment combining chemo- and radiotherapy, NPC presents high metastatic rates and common recurrence. Despite advancements in immune-checkpoint inhibitors (ICIs) and cytotoxic-T-lymphocytes (CTLs)-based cellular therapy, the exhaustive T cell profile and other signs of immunosuppression within the NPC tumour microenvironment (TME) remain as concerns to immunotherapy response. Exosomes, extracellular vesicles of 30–150 nm in diameter, are increasingly studied and linked to tumourigenesis in oncology. These bilipid-membrane-bound vesicles are packaged with a variety of signalling molecules, mediating cell–cell communications. Within the TME, exosomes can originate from tumour, immune, or stromal cells. Although there are studies on tumour-derived exosomes (TEX) in NPC and their effects on tumour processes like angiogenesis, metastasis, therapeutic resistance, there is a lack of research on their involvement in immune evasion. In this review, we aim to enhance the comprehension of how NPC TEX contribute to cellular immunosuppression. Furthermore, considering the detectability of TEX in bodily fluids, we will also discuss the potential development of TEX-related biomarkers for liquid biopsy in NPC as this could facilitate early diagnosis and prognostication of the disease.

## 1. Introduction

Nasopharyngeal carcinoma commonly originates in the nasopharyngeal mucosal epithelium of the posterolateral nasopharynx and the fossa of Rosenmüller. It has a high prevalence in Southeast Asia and Africa [1]. Despite the current therapeutic approach combining chemo- and radiotherapy, therapeutic resistance and the occurrence of metastatic relapse remain as significant challenges in the treatment of NPC [2]. Due to its unique location and absence of specific early symptoms, NPC is typically diagnosed at an advanced stage, further impacting the prognosis [3]. Therefore, there is a need to explore suitable drugs and novel biomarkers to enhance the prevention, detection, and therapy of NPC.

Liquid biopsy is a non-invasive diagnostic or prognostic approach that holds great promise, particularly for NPC, from which obtaining tissue samples can be challenging. Given the close relationship between Epstein–Barr virus (EBV) and NPC, circulating EBV DNA has been explored as a tumour marker for NPC. Currently, screening methods for NPC include serum EBV-related antibody assays and EBV DNA tests [4]. However, these approaches have limitations when it comes to diagnosing patients with early-stage NPC. Serum EBV-related antibodies testing is only effective on EBV-positive NPC patients, while EBV DNA has low sensitivity in early-stage patients due to low tumour volume. The presence of other EBV-related non-NPC diseases such as nasopharyngitis also hampers the specificity [5,6]. Non-liquid screening methods such as endoscopy and tissue biopsies, on the other hand, are invasive and only available for patients with no contraindications to undergo such procedures [3]. The need for liquid-based diagnostic biomarkers sensitive to early NPC is therefore imperative.

Extracellular vehicles (EVs) are membrane-bound nanoparticles naturally released from cells [7]. The classification of EVs can be size based, with 200 nm as the cutoff differentiating small EVs from medium/large EVs; or origin based—whether the EVs are derived from cell plasma membrane or endosome [8,9]. Exosomes, unlike plasma membrane-derived medium/large EVs, are small EVs that range in size from 30 to 150 nm [9]. Exosomes originate from the subcellular component multivesicular endosome, and are released from cells upon fusion of multivesicular bodies with the plasma membrane [9]. During their biogenesis, exosomes are packaged with proteins, lipids, DNAs, messenger RNAs (mRNAs), and other non-coding RNAs such as microRNAs (miRNAs) [10]. miRNAs are known inhibitors of mRNAs translation, thereby regulating gene expression. Along with other proteinaceous signalling molecules, the uptake of exosomes by target cells would result in cellular changes in terms of cell cycle regulation, differentiation, migration, stress response, and more [10].

All cells, including healthy, diseased, or even malignant cells, can release exosomes into extracellular spaces—blood plasma, saliva, urine, breast milk, ascitic, amniotic and cerebrospinal fluid [9]. EBV-infected NPC cells, in particular, have demonstrated increased secretion of exosomes, as prompted by the oncogenic EBV latent membrane protein 1 (LMP-1) [11,12]. Tumour-derived exosomes (TEX) are first released into the tumour microenvironment (TME), which is the immediate surroundings of the tumour. Interactions between TEX and immune/stromal cells within the TME may eventually contribute to tumour growth, immune evasion, angiogenesis, metastasis, and therapeutic resistance, as iterated in other cancers [13,14,15]. Apart from exosomes, tumour cells can also release other EVs to modulate the TME. Microvesicles (MVs), for instance, are nanoparticles of 150 to 1000 nm in diameter, formed by the outward budding of the plasma membrane [16,17]. Studies have shown that tumour-derived MVs can also activate fibroblasts, stimulate angiogenesis, and mediate immune evasion [18,19,20,21]. Apoptotic bodies, EVs released from dying cells but significantly larger than exosomes or MVs, have also been recently indicated in cancer biology for their value in promoting anti-tumour immunity and enhancing diagnosis [22]. However, considering the great diversity of EVs, we will be mainly focusing on exosomes, which are better studied in the field of NPC.

NPC, as a cancer characterised by EBV latent infection and the constant expression of oncogenic EBV proteins, is indivisible with inflammation and immune cell infiltration [23,24]. Yet, the TME of NPC is immunosuppressed, and the mechanisms underlying immune evasion remain poorly understood [25]. The immune landscape of NPC has only started to become clearer to us with the recent advancements in single-cell and spatial transcriptomics [24]. NPC-derived exosomes (NPC-TEX) may play a crucial role in regulating immune signalling cascades within the TME or the tumour immune microenvironment (TIME), thereby mediating immune evasion. However, the exact mechanisms through which NPC-TEX influence the TME and contribute to immune suppression require further elucidation. Additionally, given the presence of TEX in virtually all body fluids, TEX carrying NPC-specific markers can be detected in liquid biopsy and may allow for early cancer detection and disease monitoring [26]. The rising understanding of exosome-mediated pathogenic pathways in NPC also presents us with novel drug targets and the potential of exosome-based drug delivery systems.

**Figure 1:**
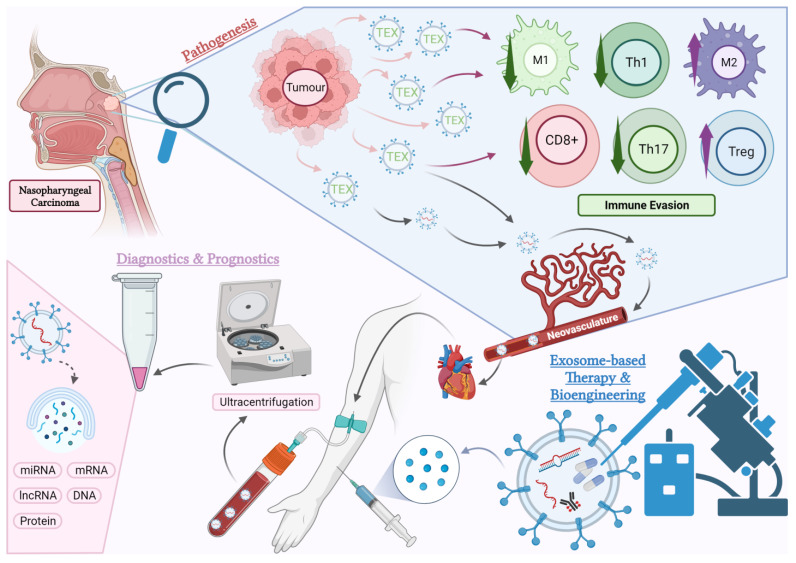
An overview of the aspects of exosome research covered in this article. Pathogenesis: Tumour-derived exosomes (TEX) mediate numerous pathogenic pathways causing immunosuppression in the tumour microenvironment (TME) of NPC. The effects of TEX on certain subtypes of immune cells within the TME such as cytotoxic T cells (CD8+), helper T cells (Th), regulatory T cells (Tregs), M1 and M2 macrophages will be the focus of this review. Diagnostics and Prognostics: TEX enter the circulation via neovasculatures in the tumour and can be detected in blood plasma and other body fluids. These exosomes carry tumour-specific molecules, which have the potential to serve as predictive or diagnostic biomarkers. Current research is exploring the use of exosome-based liquid biopsies in NPC. Treatment: Exosomes derived from the host or artificially manufactured can be bioengineered and used as therapeutic agents. This opens new possibilities for future pharmacological studies in NPC (created with BioRender.com, accessed on 23 October 2023).

In this review, we aim to comprehensively discuss the clinically significant aspects of exosome research in NPC (Figure 1). We will start by discussing the intricate network between NPC-TEX and immune cells. Further, we will highlight the clinical potential of exosomes as targets for therapy, therapeutic agents themselves, as well as diagnostic and prognostic biomarkers for NPC. Finally, we will outline and discuss future research directions for exosomes in NPC along with the limitations.

## 2. Exosomes in the Regulation of NPC Immune Response

TEX have a significant impact on anti-tumour immunity [27,28,29]. They can impede the function of immune cells such as T cells and dendritic cells (DCs) [30,31,32], preventing them from being activated or inducing their dysfunction. Additionally, TEX can also stimulate the recruitment and production of immunosuppressive cells, such as regulatory T cells (Tregs) and myeloid-derived suppressor cells (MDSCs) [33,34], further weakening the immune response against cancer. Therefore, understanding the mechanisms behind exosome-mediated immunosuppression is vital for developing innovative strategies that enhance anti-tumour immunity and ultimately improve cancer treatment outcomes. In this section, we shall focus on the effects of TEX on macrophages and T cells.

### 2.1. TEX Contribute to the Differentiation of Macrophages into Highly Immunosuppressive 129 M2-Polarised Phenotype

Innate immunity involves various immune cells. Macrophages, in particular, constitute the majority of the inflammatory infiltrate in TMEs, both in terms of abundance and functionality [35]. Given the functional plasticity of macrophages, they polarise into different functional phenotypes in response to different environmental stimuli—M1 (anti-tumour)/M2 (pro-tumour) [36]. In most cancers, macrophage-infiltrates within the TME, termed tumour-associated macrophages (TAMs), are skewed towards the M2 phenotype and are associated with the promotion of tumour progression and survival [37]. They are known for stimulating angiogenesis, fostering an inflammatory milieu, and suppressing anti-tumour immunity in the TME via altered cytokine profiles [38,39].

Macrophage migration inhibitory factor (MIF) was suggested to play a predominant role in M2 macrophage polarisation, and MIF depletion spontaneously reverts TAMs to an M1-like polarisation, as found in oral squamous cell carcinoma [40]. Chen et al. subsequently reported that MIF was upregulated in NPC and was related to a lower survival rate [41]. More importantly, they have located MIF in exosomes derived from NPC cells. These MIF-rich TEX will not only affect the polarisation of M2 macrophages through the AMP-activated protein kinase (AMPK) pathway, but also prolong the survival of tumourigenic TAMs by reducing their ferroptosis through the upregulation of glutathione peroxidase 4 (GPX4) [41]. Following, another study has connected NPC-TEX-mediated macrophage polarisation to the degradation of phosphatase and tensin homolog (PTEN) in TAMs [42]. PTEN instability has also been previously reported to cause bladder cancer progression [43]. The ring finger protein 126 (RNF126) found in NPC-TEX was shown to promote PTEN ubiquitination and, thus, degradation, in the exosome-receiving TAMs [42]. Consequently, the PI3K/AKT pathway will be activated, and autophagy will be inhibited, resulting in increased polarisation, migration, and invasion of TAMs [42]. Autophagy was reported to be an important defence against M2 polarisation [44,45,46]. Therefore, it is conceivable that RNF126 in NPC-TEX contributes to the immunosuppressive TME by suppressing autophagy and promoting M2 polarisation [42]. In addition, long non-coding RNAs (lncRNAs), such as the tumour protein P73 antisense RNA 1 (TP73-AS1), have also been shown to take part in TEX-mediated M2 polarisation [47]. Yao et al. reported that tumour protein P73 antisense RNA 1 (TP73-AS1) is upregulated in NPC and associated with a poorer prognosis [47]. Their further investigation showed that TP73-AS1 delivered by TEX could lead to M2 polarisation of macrophages and alter their cellular functions. To explain the phenomenon, one possible target of this lncRNA is the tumour-suppressor miR-342-3p. TP73-AS1 has been demonstrated to have a negative correlation with miR-342-3p, possibly due to its ability to directly bind to and sponge miR-342-3p [47].

Functional changes in TAMs, in terms of phagocytosis and cytokine production, can also be affected by NPC-TEX [48,49]. A recent study on scavenger receptor class B member 1 (SCARB1) reported its enrichment in NPC-TEX, and these TEX can be taken up by macrophages to confer tumourigenicity by targeting the transcription factor krueppel-like factor 9 (KLF-9) [48]. In M1 macrophages, SCARB1 upregulates the expression of 3-hydroxyanthranilate 3,4-dioxygenase (HAAO), leading to ferroptosis of the macrophages [48]. This reduces the protective effects from the M1 subtypes. Likewise, in M2 TAMs, SCARB1 downregulates the CYP1B1 gene of the cytochrome P450 family, inhibiting their phagocytic capacity [48]. As a result, the remnant anti-tumour effects of M2 TAMs will also be hampered. Wang et al., on the other hand, found increased IL-6 secretion in macrophages treated with NPC-TEX, which is potentially linked to tumour progression and immunosuppression [49]. Chronic inflammation in the TME is known to promote tumour progression [50]. The linkage between EBV infection, immunity, inflammation, and NPC is largely contributed by the cytokines produced from the activated innate immune cells. TNF-α, TGF-β, and IL-6, -10, -12, -17, are some commonly studied examples [51]. IL-6, in this case, has shown capability to induce NPC growth via STAT3 activation and IL-6R overexpression in the EBV-infected epithelial cells [52]. In addition, IL-6 can also lead to immunosuppression such as by reducing the abundance of CD8+ tumour-infiltrating lymphocytes (TILs) in the TME while expanding the Tregs population, as demonstrated in other cancers [53].

In short, NPC-TEX alter TAMs phenotypically and functionally, resulting in an immunosuppressive TME. This highlights the significance of TEX in NPC immune evasion. Future studies may consider investigating the possible effects of TEX on other innate immune cells more in depth as well. On the other hand, the mechanisms underlying the cytokine changes in TAMs, such as that of IL-6, can also be further studied [49]. As such, this will help us to map out how TEX orchestrate the inflammatory milieu in NPC. By understanding the pathogenic effects of this network, potential therapeutic targets can also be identified for controlling it.

### 2.2. Exosome-Mediated Inhibition of T Cells in NPC TME

Another typical feature of the NPC is the high abundance of TILs in its TME, with a rich CD8+ profile [54,55]. Paradoxically, the NPC TME is also known to be immunosuppressive, with poor effector cell function, characterised by exhaustive markers [56]. The presence of abnormal quantities of Tregs within the tumour site and the peripheral blood is both an explanation and a clear indication of it [57]. Exosomes as insidious regulators in the TME may be implicated in this exhaustive T cell profile.

#### 2.2.1. TEX-Mediated T Cell Inhibition Mechanisms Associated with EBV

Since NPC is associated with EBV infection, the interplay between EBV+ NPC cells and TILs has always been an area of focus. Exosomes derived from EBV+ tumour cells might therefore have implications for T cell exhaustion. Galectin-9 is a highly abundant protein in NPC, and is associated with exosomes [58]. It is a known agonist for the T-cell immunoglobulin and mucin domain (Tim-3), which is a death-inducing receptor expressed on mature Th1 cells [59]. Researchers specifically studied the effects of galectin-9 in exosomes derived from EBV+ NPC cells and validated their capability in inducing Th1 apoptosis through galectin-9/Tim-3 interaction [60]. However, it should be noted that Th1 cells in the in vitro study were derived from healthy EBV carriers instead of NPC patients. Whether Th1 in the actual NPC TME would express the same level of Tim-3 and undergo apoptosis is yet to be further confirmed in future studies [60]. Following, cancer-associated fibroblasts (CAFs), are also linked to exosomes [61]. CAF is a population of cells involved in extracellular matrix (ECM) remodelling, and potentially, immunosuppression [62,63]. Reported in a study, TEX containing EBV products, such as LMP-1, were found to activate the yes-associated protein (YAP1) signalling pathways in fibroblasts, leading to the formation of CAFs [61]. These CAFs had an increased secretion of immunosuppressive cytokines IL6, IL8, and CCL2 [61]. Through 3D spheroid model studies, it was confirmed that these TEX-induced CAFs would reduce the abundance of CD8+ T lymphocytes in TME [61]. While EBV was mentioned in these studies, the comparison with EBV- NPC cells-derived TEX was inadequate, and the evidence may not be conclusive. A panoramic understanding of the role of EBV in TEX-mediated immune suppression is yet to be drawn.

#### 2.2.2. miRNAs in NPC-TEX Alters the Differentiation Patterns and Secretome of T Cells

The suppression of NPC-TEX on the activation and proliferation of both CD4+ and CD8+ T cells has also been attributed to several miRNAs—“miR-24-3p, miR-20a-5p, miR-891a, miR-106a-5p, and miR-1908” [30]. miR-24-3p, in further investigations, was found to target fibroblast growth factor 11 (FGF11) [64]. miR-20a, denoted by another study, also affects STAT3 signalling [65]. FGF11 was recognised as the central regulator in the mitogen-activated protein kinase (MAPK) pathway [64]. The suppression of FGF11 by miR-24-3p, alongside the effects of other miRNAs in the TEX, disrupted the MAPK signalling in T cells. This resulted in altered phosphorylation of ERK and STAT proteins in the downstream [64]. Consequently, the differentiation of CD4+ T cells into Th1 and Th17 cells was inhibited, while their differentiation into Tregs was promoted [30]. This might, in turn, hamper the activity of CD8+ T cells. IFN-γ, as indicated by inflammatory studies, is not only an important activator of effector T cell, but also an antagonist to Tregs’ proliferation and function. The depletion of IFN-γ-producing Th1 cells could explain the diminished CD8+ effector functions and the expansion of Tregs within the TIME of NPC [66]. Th1 differentiation itself has also demonstrated inhibitory effects on Tregs’ generation in specific contexts [67]. The function is, however, likely compromised in NPC. Subsequent to phenotypical changes, the cytokine profile of T cells would also be affected. CD4+ T cells were shown to produce more IL-6 but less IL-2, IL-12, IL-17, and IFN-γ [30]. Likewise, CD8+ T cells also showed increased production of IL-6 and IL-10 but reduced TNFα [30]. The roles of IL-6 in tumour progression and immunosuppression have been discussed [52,53], and the immunostimulatory roles of cytokines like IFN-γ, IL-2, and TNFα in cancer biology are also known [68,69]. NPC-TEX, in short, increases immunosuppressive cytokines and reduces immunostimulatory cytokines within the TME, further contributing to immune evasion.

#### 2.2.3. NPC-TEX Directly Recruit Tregs to Promote Immune Evasion

The expansion of Tregs in the NPC TME could not only be secondary to the phenotypical and secretome changes in T cells, but also occur under the direct effects of TEX. Mrizak et al. unveiled that NPC-TEX could directly recruit Tregs into the TME and promote their proliferation via CC motif chemokine ligand and receptor (CCL20-CCR6) interaction [33]. In further investigations, NPC-TEX were also found to recruit conventional CD4+ CD25- T cells into the TME and promote their differentiation into CD4+ CD25^high^ Tregs [33]. These TEX-induced Tregs presented with an altered cytokine profile in which they tend to secrete more IL-10 and transforming growth factor beta 1 (TGFB1), further contributing to an immunosuppressive TME [33].

#### 2.2.4. NPC-TEX Surface Proteins Contribute to T Cell Exhaustion

The latest studies on NPC-TEX tend to focus more on improving tumour sensitivity to immune checkpoint inhibitors (ICI), which is highly relevant to T cells [70]. It is known that tumour cells and their exosomes express surface PD-L1 (Exo-PD-L1), contributing to immune evasion [71]. Yang et al. validated in NPC that Exo-PD-L1 could substantially inhibit the proliferation, cytokine production, and tumour infiltration of CD8+ cytotoxic T lymphocytes (CTLs) [31]. Such immunosuppression could be reversed by anti-PD-L1 antibodies, or exosome secretion inhibitors, presenting potential avenues in immunotherapy in addition to the current use of anti-PD-1 antibodies such as pembrolizumab and nivolumab [72]. Moreover, a recent study on melanoma discovered that the loading of PD-L1 into TEX is mediated by the phosphorylation of hepatocyte-growth-factor-regulated tyrosine kinase substrate (HRS), a key component of the machinery which regulates the sorting of cargos into exosomes, suggesting that specifically inhibiting the phosphorylation of HRS in tumour cells could enhance the efficacy of ICI treatments [73].

#### 2.2.5. Potential Pathways of TEX-Mediated T Cell Inhibition Yet to Be Found in NPC

Exosome-mediated suppression of T cells can also take place via indirect routes. CTLs’ activation and proliferation depend largely on antigen-presenting cells (APC) MHC I. Therefore, alterations in DCs’ maturation and antigen presentation are plausible causes of CTLs inhibition. Early in 2013, researchers pointed out that TEX can block the differentiation of myeloid precursors and dendritic cell precursors, resulting in a reduction in DCs and expansion of MDSCs [74]. Likewise, S100A9 in melanoma TEX reduced the expression of CD83, CD86, IL-12, and IL-15 in DCs, inhibiting their maturation [75]. Moreover, TEX galectin-9 in glioblastoma inhibited the maturation of DCs and prevented antigen presentation, hampering the activation of CTLs in the cerebrospinal fluid [76]. TEX in a prostate cancer model also exhibited a predominant role in inducing a non-native CD73+ phenotype in DCs, promoting an adenosine-mediated suppression of CD8+ T cell activation via impaired DC antigen presentation [32]. Similar exosome-mediated mechanisms that indirectly result in the inhibition of T cells or other immune cells are, however, poorly understood in NPC. Given the central role of CTLs in immunotherapy, it is suggested that related pathways should be uncovered.

**Figure 2:**
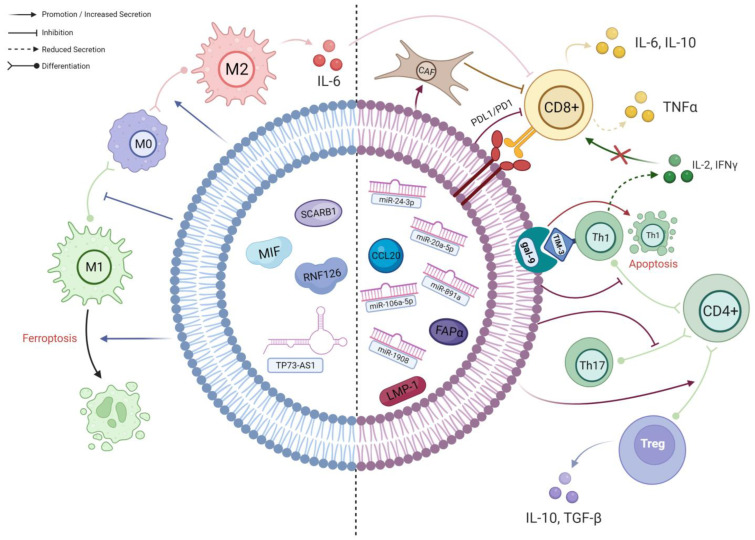
A schematic diagram of the known interactions between TEX and different immune cells in NPC. The **left side** of the TEX (BLUE) shows cargos which predominantly affect macrophages; their effects are mainly on macrophage polarisation and viability. The **right side** of the TEX (RED) demonstrates the cargos involved in the complex interplay between different subpopulations of T cells. As delineated, CD4+ T cells preferentially differentiate into immunosuppressive Tregs under the influence of TEX, resulting in an altered cytokine secretion profile. Along with the effects of other TEX cargos, the infiltration and activation of CD8+ T cells will ultimately be hampered, leading to further immune evasion (created with BioRender.com, accessed on 22 October 2023).

To summarise, TEX impede the anti-tumour response of T cells through numerous mechanisms, which can potentially diminish the efficacy of immunotherapies. By counteracting TEX, we could potentially enhance the success of immunotherapy in treating NPC (Figure 2).

### 2.3. Involvement of Exosomes in the Disease Progression and Metastasis of NPC

Tumour progression is the summed outcome of numerous processes. Apart from immune evasion, events such as angiogenesis, epithelial-to-mesenchymal transition (EMT), therapeutic resistance, etc., also play indispensable roles [77]. Through these processes, the cancer becomes more aggressive in clinical behaviour over time [78], resulting in increased tumour growth, invasiveness, and ultimately metastasis [79]. Accumulating evidence shows that TEX might be involved in facilitating these processes. Here, we aim to provide several examples to further highlight the importance of exosome studies in NPC (Table 1).

## 3. Potential of Exosome-Based Therapy Targeting Immunosuppression in NPC

Exosomes themselves stand as promising vehicles for the delivery of drugs or anti-cancer molecules, as they are protected from macrophage phagocytosis through a CD47-mediated pathway [97,98]. Additionally, exosomes are equipped with adhesion and signalling molecules that facilitate their binding to and uptake by their destined cells [99]. These unique properties, coupled with their high scalability, bioavailability, and biocompatibility, render exosomes a highly efficient therapeutic option. They surpass free-drug or cell-based therapies in this regard, which are typically more vulnerable to immune clearance [100,101].

Whilst some researchers in the realm of NPC have started investigating the possible use of exosome-based strategies to reverse tumour processes such as angiogenesis, metastasis, and therapeutic resistance, there has been little research on reversing immunosuppression (Table 1). The following paragraphs, therefore, delve into the recent advancements in exosome-based immunotherapy in other cancers and the potential of their incorporation into NPC treatment. Since the TIME of NPC is similar to that of the cancer types to be mentioned [102,103], except for some minor subpopulations such as double negative B cells [104], we hypothesise the potential development of similar therapeutic strategies in NPC. Below, we present a summary of the current exosomal strategies identified to treat NPC to further illustrate the plausibility of this direction (Table 2).

### 3.1. Exosomes Carrying Specific Antigens Can Be Developed as Cancer Vaccines

One of the current exosome-based strategies to improve anti-tumour immunity is to use exosomes in cancer vaccines. A cancer vaccine is a treatment which introduces tumour-associated antigens (TAAs) into humans to educate the immune system to specifically recognise and kill the cancer cells [105]. DCs, cells responsible for presentation of TAAs to trigger adaptive immunity, are involved in most of the latest strategies. Previous evidence showed that after taking up TAAs, DCs would package the MHC-antigen complex, along with other immunostimulatory factors into exosomes [106]. These DCs-derived exosomes (DEX) could transfer the MHC-antigen complex to other inactive DCs in peripheral lymph nodes and boost their ability to activate T cells [106]. Morse et al. subsequently conducted a phase I clinical trial on the use of DEX in enhancing an anti-tumour immune response. They carried out leukapheresis on non-small-cell lung cancer (NSCLC) patients to retrieve autologous DCs as continuous sources of DEX. The DEX were then loaded with melanoma antigen gene (MAGE), a type of tumour antigen, and administered back into the patients at four doses per week. The vaccine was well tolerated by advanced patients, and increased NK lytic activity was observed in half of them [107].

Apart from DEX, TEX may also prove to be effective agents for cancer vaccines due to the inherent presence of TAAs in these [108,109]. Researchers, in this regard, attempted to harvest, modify, and then reintroduce TEX into cancer patients to enhance DCs+ antigen presentation and immune activation in vitro and in vivo. Colon carcinoma TEX, when augmented in the expression of IL-12 and a shRNA (short hairpin RNA) targeting TGF-β1, compared to unmodified TEX, showed higher efficacy in tumour growth inhibition and immune activation in conjunction with a DC-based vaccine [110]. In melanoma, TEX have been biologically engineered to present immunostimulatory CpG-DNA such that they are optimally taken up by DCs [111]. Likewise, melanoma TEX added with pH-sensitive fusogenic GALA peptide also improved DCs’ antigen recognition [112]. In other studies on hepatocellular carcinoma (HCC), TEX incorporated with other molecules such as miR-155 and high-mobility group nucleosome-binding protein 1 (HMGN1) have also improved DCs’ maturation and antigen presentation [113,114]. Further downstream investigations have also proven that the enhancement of DCs using TEX would eventually result in the reinvigoration of T cells [113,114]. Hence, TEX can be bioengineered and employed in cancer vaccines to enhance the capability of DCs to obtain and present TAAs. This leads to an enhanced adaptive immune response against cancer cells.

Yet, in the context of NPC, the direction of an exosome-based cancer vaccine remains unexplored. As previously discussed, NPC-TEX are embodied with T cell inhibitory molecules such as LMP-1 and PD-L1 [31,61]. Also, though not studied yet in NPC, TEX carrying cargos like S100A9 and galectin-9 have also shown the ability to reduce DCs’ maturation and antigen presentation in other cancers [75,76]. TEX carry a variety of pro-tumour and immunosuppressive molecules. When delivered to the wrong cells, it may result in dire consequences, especially for NPC-TEX, where there are also EBV oncogenic proteins. As of now, bioengineering techniques for the removal of specific TEX content such as LMP-1 and PD-L1 have not been developed [115]. Intriguingly, it has been reported that DEX exhibit a more potent ability to activate CTLs compared to TEX when used in cancer vaccines [110,112,114]. Therefore, DEX might stand as a more promising direction than TEX in future NPC therapeutics’ development.

### 3.2. Drug Delivery via Bioengineered Exosomes Can Modulate Anti-Tumour Immunity

Exosomes can also be utilised as drug or molecule delivery system to directly reverse immunosuppression or induce an immune response given their stability and cancer-cell-targeting efficacy. Zhou et al. developed a dual delivery system for pancreatic cancer using bone marrow mesenchymal stem cell (BM-MSC)-derived exosomes [116]. They loaded the exosomes with galectin-9 siRNA (small interfering RNA) through electroporation, followed by modification of exosome surface with oxaliplatin (OXA) prodrug [116]. The combined therapy successfully disrupted the galectin-9/dectin-1 axis to promote M1 polarisation in macrophages and triggered immunogenic cell death (ICD) to recruit CTLs [116]. Another study on renal cell carcinoma (RCC) also revealed that genetically modifying human RCC cells to express IL-12 resulted in TEX co-expressing TAAs and IL-12 [117]. These TEX can efficiently induce antigen-specific CTLs, promote their proliferation, and increase their release of IFN-γ [117].

Although the use of bioengineering to develop immunotherapeutic agents remains undeveloped in NPC, the increasing body of evidence from other types of cancer research is calling attention to this possibility. Stimulator of interferon genes (STING), an endoplasmic reticulum transmembrane protein, can be packaged into TEX. These STING-rich TEX could recruit tank-binding kinase 1 (TBK1) to activate interferon regulatory factor 3 (IRF3) in recipient cells [118]. This induces the production of various cytokines, including type I interferons (IFNs). It has been demonstrated that activated STING transferred by TEX could induce IFNβ production in macrophages, which can then recruit CD3+ and CD8+ T cells into the TME [118]. miR-6750 is a microRNA intrinsically present in NPC-TEX but downregulated compared to the levels found in other small extracellular vesicles (SEVs) and in the serum of normal people [119]. Manual upregulation of miR-6750 in NPC cells increased its level in TEX. These miR-6750-rich TEX have demonstrated anti-tumour capacity by promoting M1 polarisation in macrophages and inhibiting angiogenesis [119]. From a clinical point of view, bioengineered exosomes enriched in miR-6750 could potentially be an adjuvant to immunotherapy considering their ability to reverse the immunosuppressive effects of TAMs. STING-loaded exosomes or exosomes packaged with STING-agonists as therapeutic agents in NPC are also feasible options for improving the tumour infiltration of T cells. However, further efforts are required to ascertain the effectiveness of such agents in NPC, as well as to identify other potential molecules that could be leveraged in exosome delivery systems for NPC treatment.

Furthermore, Shi et al. have recently introduced a pioneering concept termed synthetic multivalent antibodies retargeted exosome (SMART-Exo) [120]. This was achieved by genetic manipulation of endogenous exosomes to simultaneously exhibit two clusters of monoclonal antibodies on their surfaces. In an experimental setup involving human epidermal growth factor receptor 2 (HER2)-positive breast cancer, the researchers engineered exosomes to display both anti-human CD3 and anti-human HER2 antibodies. This provided the SMART-Exos with the capacity to concurrently attach to T lymphocytes and HER2+ breast cancer cells. The SMART-Exos demonstrated significant potency both in vitro and in vivo in guiding CTLs to specifically target HER2+ breast cancer cells [120]. This novel technique underscores the possibilities in exosome-based immunotherapy and, ideally, provides valuable insights that future research in NPC could draw upon.

### 3.3. Naturally Occurring Exosomes Can Be Extracted and Utilised in Immunotherapy

The use of naturally occurring exosomes derived from the host’s immune cells also presents a potential avenue for immunomodulation. Several studies have delved into the potential use of exosomes derived from M1 macrophages (M1-EX) as adjuvants in immunotherapy. Research conducted on colon carcinoma demonstrated that M1-EX could effectively repolarise M2 macrophages to M1 macrophages, both in vitro and in vivo [121]. Moreover, a combined usage of M1-EX and PD-L1 inhibitors demonstrated a more significant reduction in tumour size in vivo than the administration of either agent alone [121]. A separate study investigating the application of M1-EX in melanoma found that following subcutaneous injection, M1-EX were preferentially trafficked to lymph nodes and absorbed by local macrophages and dendritic cells (DCs) [122]. This led to a predominant polarisation of macrophages to the M1 phenotype, which secreted pro-inflammatory cytokines consistent with a Th1 profile, including IL-6, IL-12, and IFN-γ. Similar effects were observed in DCs. Furthermore, the study found that M1-EX, when used in conjunction with a lipid calcium phosphate (LCP)-encapsulated tyrosinase-related protein-2 (Trp2) vaccine, induced a stronger antigen-specific CTLs response [122]. This effect also proved superior to other vaccine potentiators, such as CpG oligonucleotide [122]. These findings suggest that M1-EX has the potential to counteract the immunosuppressive effects of TAMs in various cancers. In addition, as an adjuvant, M1-EX could also enhance the efficacy of other immunotherapies, like ICI and cancer vaccines, or even that of chemotherapy.

Another area of interest within this field pertains to NK cell-derived exosomes (NEX). Zhu L et al. initially demonstrated that NEX naturally contain molecules like perforin, FasL, and TNF-α, which enable them to directly induce apoptosis in melanoma cells [123]. Subsequently, they engineered nanovesicles that mimic NEX by passing NK cells through filters with progressively smaller pore sizes, creating entities known as NK-EM [124,125]. Interestingly, NK-EM exhibited higher cytotoxicity than NEX when tested against glioblastoma, breast carcinoma, anaplastic thyroid cancer, and hepatic carcinoma [126]. Further efforts indicated that priming the NK cells with IL-15 before NEX extraction could also enhance tumour targeting and cytotoxicity of NEX, thus expanding their potential for use in immunotherapy [123]. Recent research has validated the potential role of NEX in cancer treatment by examining cytotoxic molecules on NEX such as DNAX accessory molecule-1 (DNAM1) and demonstrating the anti-tumour effect of NEX in NSCLC [124,125]. In relation to NPC, a recent study showed that exosomes isolated from γδ-T cells (γδ-T-Exos) could augment the expression of CCR5 on host T cells [126]. Coupled with the abundant secretion of CCL5 from NPC cells, γδ-T-Exos can foster T cell infiltration into the NPC TME. Consequently, this approach holds the potential to enhance NPC response to adoptive cytotoxic T-cell-based therapy and other forms of immunotherapy [126].

### 3.4. Exosome-Based Strategies to Reverse Other NPC Tumour Processes

Presently, multiple pathways contributing to oncogenic processes like tumour angiogenesis, permeabilisation of blood vessels, formation of vasculogenic mimicry (VM), epithelial-to-mesenchymal transition (EMT), and resistance to therapy in NPC have been identified. Investigating the suitability of exosomes as delivery vehicles, researchers in the field have attempted to load exosomes with specific anti-tumour cargos to target those pathways. The bioengineered exosomes can be of artificial origin, derived from healthy mesenchymal stem cells (MSCs), or derived from host NPC-TEX. Meanwhile, the direct usage of natural, unmodified exosomes from other cells such as γδ-T cells has also been studied. As of now, effective exosome-based therapeutic agents to reduce vascularisation, inhibit EMT, and improve chemo-/radiotherapy have been unveiled (Table 2).

**Table 2:** Novel exosome-based treatment agents targeting NPC tumour processes.

Treatment Effect(s)	Exosome Content(s)	Target Gene(s)/Pathway(s)	Mechanism of Action	Ref.
Inhibits Vasculature Formation	miR-9	MDK, PDK/AKT	miR-9, as a strong tumour suppressor, suppresses endothelial tube formation and migration by downregulating the MDK gene and repressing the MDK/AKT pathway.	[127,128]
	miR-125a	TAZ	miR-125a inhibits the migration and formation of vasculogenic mimicry in NPC cells.	[128,129]
	antagomiR-BART10-5p, antagomiR-18a	EBV-miR-BART10-5p, hsa-miR-18a	Pro-angiogenic miRNAs EBV-miR-BART10-5p and pro-metastatic hsa-miR-18a can be inhibited by their corresponding antagonists to slow tumour progression via downstream Spry3 pathway.	[130]
	antagomiR-BART1-5p	EBV-miR- BART1-5p	EBV-miR-BART1-5p promotes vasculogenic mimicry formation and angiogenesis through downstream Spry2/AMPK/mTOR/HIF1 pathway. Its antagonist reverses the effects.	[131]
VEGF Inhibition	LBH	CRYAB	LBH inhibits VEGFA expression in NPC cells and ECs. Inhibition of EMT in NPC cells was also observed.	[132]
	Guggulsterone	circFIP1L1	Guggulsterone promotes the secretion of exosomal circFIP1L1 from NPC cells, which sponges miR-125a-5p—a highly expressed miRNA in NPC tissues and cells that promotes angiogenesis through VEGFA upregulation.	[133]
Inhibits Metastasis	Aspirin	LMP-1	Trafficking of LMP-1, an EMT-promoting molecule, into NPC-TEX was found to be NF-kB dependent. Aspirin as an NF-kB inhibitor hampers the process, thus depleting the TEX from LMP-1.	[134]
	miR-6750	M6PR	miR-6750 attenuates metastasis by inhibiting angiogenesis and promoting M1 macrophage polarisation to reduce the pro-metastatic effects of M2 TAMs.	[119]
	miR-34c	β-Catenin	miR-34c inhibits invasion, migration, proliferation and EMT of NPC cells.	[135]
	γδ-T-exosomes, unspecified content	Fas/FasL, TRAIL/DR5, CCR5	γδ-T-exosomes directly induce apoptosis in NPC cells, and, more importantly, CSCs via Fas/FasL and TRAIL/DR5 interactions. They also facilitate T cell migration into TME by upregulating their expression of CCR5, further limiting the expansion of NPC cells. When combined with irradiation, γδ-T-exosomes also concentrate more in the TME, showing a synergistic effect.	[126]
Improving Chemo-/Radiotherapy	miR-197-3p	Akt/mTOR	For refractory NPCs, they are often resistant to intensive treatment including radiotherapy. miR-197-3p can serve as a radiosensitiser and therapeutic agent by inhibiting AKT/mTOR activation and HSPA5-mediated autophagy.	[136]
	miR-142-5p	HGF/c-Met, EGF/EGFR	miR-142-5p inhibits both HGF/c-Met and EGF/EGFR pathways to restore radiosensitivity in resistant cells.	[137]
	miR-34c	β-Catenin	miR-34c reduces radioresistance by specifically suppressing β-catenin, thus increasing apoptosis of NPC cells under irradiation.	[135]
	miR-183-5p	MDR1	miR-183-5p targets MDR1 and can reduce P-glycoprotein expression (efflux pump) in paclitaxel-resistant NPC cells.	[138]

Abbreviations: “AKT: protein kinase B; AMPK: AMP-activated protein kinase; EBV-miR-BARTs: Epstein–Barr virus-encoded BamHI-A Rightward Transcript microRNAs; CCR5: C-C motif chemokine receptor 5; CSCs: cancer stem cells; CRYAB: crystallin alpha B; EGF: epidermal growth factor; EGFR: epidermal growth factor receptor; EMT: epithelial-mesenchymal transition; FasL: Fas ligand; HGF: hepatocyte growth factor; HIF1: hypoxia-inducible factor 1; HSPA5: heat shock protein family A (Hsp70) member 5; LBH: limb bud and heart gene; LMP-1: latent membrane protein 1; M6PR: mannose-6-phosphate receptor; MDK: midkine; MDR1: multi-drug resistance protein 1; mTOR: mammalian target of rapamycin; PDK: pyruvate dehydrogenase kinase; Spry: sprouty RTK signalling antagonist; TAZ: tafazzin; TRAIL: TNF-related apoptosis-inducing ligand; VEGF: vascular endothelial growth factor”.

## 4. Developing TEX as Predictive or Diagnostic Biomarkers for NPC

The concentration and composition of bioactive molecules within TEX can change with the physiological or pathological state of the originating tumour cells, making their analysis a valuable tool for assessing the presence and status of tumour cells [139].

Recent advancements in the field of liquid biopsy, which enables the molecular examination of fluid samples, most commonly blood, have expanded the range of detectable biomarkers [140]. Conventionally, this approach focused on cell-free DNA (cfDNA) and circulating tumour cells (CTCs). However, it has become apparent that circulating TEX can provide additional insights into tumour cell status [26,141]. Studying TEX in liquid biopsy samples offers a minimally invasive, sensitive, and efficient method for interrogating tumour cells, in contrast to traditional tissue biopsy, which can be more invasive and less readily available. Thus, TEX-based liquid biopsies may enhance our ability to monitor tumour progression and response to therapy.

### 4.1. TEX in Predicting or Monitoring NPC Immunotherapy Response

Current NPC immunotherapy mainly focuses on PD-1 inhibitors and EBV-specific cytotoxic T lymphocytes (EBV-CTLs) [142]; this underscores the potential significance of TEX surface PD-L1 (Exo-PD-L1) and TEX-mediated T cell suppression on the effectiveness of immunotherapy.

Exo-PD-L1 is a major cause of CTLs’ exhaustion and may impede NPC immunotherapy [31]. In TEX isolated from glioblastoma and metastatic melanomas, Exo-PD-L1 demonstrated the ability to directly activate the PD-1/PD-L1 immune checkpoint [143]. Recent findings showed that Exo-PD-L1 suppressed the proliferation and tumour infiltration of CTLs in an in vivo model of NPC [31]. Exo-PD-L1 can also cause systemic immunosuppression. Researchers compared the effects of exosomes extracted from wild-type and Cd274 (PD-L1) knockdown (kd) cell lines of TRAMP-C2 prostate cancer on mouse models [144]. Mice treated with wild-type exosomes had fewer effector CTLs but more exhausted TIM3+ CD4+ and CD8+ T cells in their draining lymph nodes than those treated with Cd274-kd exosomes [144]. A higher level of Exo-PD-L1 was also associated with fewer CD4+ and CD8+ T cells and reduced granzyme B expression in gastric cancer patients, with poorer survival [145]. This association was not observed with other forms of soluble PD-L1 [145,146], possibly because exosomes express not only PD-L1 molecules but also other immune signalling molecules such as the major histocompatibility complex (MHC). PD-1/PD-L1 can only inhibit the targeted T cells when the binding happens in close proximity to MHC-TCR (T cell receptor) signalling cascades [147,148]. Compared to other soluble forms, including PD-L1 associated with other types of extracellular vesicles, PD-L1 cleaved from tumour cell surface, or other secreted forms, Exo-PD-L1 appears to hold greater immunosuppressive capacity [149,150].

Exo-PD-L1 is indeed associated with and can be a biomarker to predict NPC patients who are going to respond to immunotherapy and have better survival post treatment [31,144,151]. In our recent study, we found a connection between Exo-PD-L1 and bintrafusp alfa (M7824), a novel bifunctional treatment targeting both TGF-β and PD-L1 [151]. Initially, NPC patients with a plasma Exo-PD-L1 expression higher or lower than 3.5 pg/mL did not show significant differences in ORR (overall response rate), PFS (progressive-free survival), and OS (overall survival). However, a subset of patients exhibited changes in Exo-PD-L1 levels after beginning the treatment [151]. Specifically, patients with a change in Exo-PD-L1 level ≥ 60 pg/mL by week 4 had a significantly worse response to M7824 and poorer survival [151]. Likewise, a recent study on metastatic melanoma revealed that patients with a high pre-treatment level of circulating Exo-PD-L1 are more likely to fail to respond to PD-1 inhibitor and have a poorer clinical outcome [71]. Another study on NSCLC also reported that patients, regardless of stage, with higher levels of serum Exo-PD-L1 have poorer relapse-free survival (RPS) [152].

Circulating levels of Exo-PD-L1 can also potentially monitor the response to immunotherapy by reflecting the extent of reactivation in T cells. The same melanoma study paradoxically found that circulating Exo-PD-L1 level increases rapidly during the first 6 weeks of ICI treatment in the responders, and the increase is positively correlated with T cell reinvigoration [71]. This is likely because ICI rescues T cells and increases IFN-γ production, which, in turn, upregulates Exo-PD-L1 expression in NPC cells. Simultaneously, due to PD-1/PD-L1 blockade by ICI, increased PD-L1 would not have negative feedback on T cell activation [71]. Although increasing evidence points high Exo-PD-L1 expression during treatment in tumours to better, rather than poorer, ICI clinical outcomes, contradicting cases of good responders with low PD-L1 levels have complicated this issue [153].

In short, the pre-treatment and on-treatment levels of plasma Exo-PD-L1 can both be biomarkers for immunotherapy response but they may represent distinct messages and should be interpreted separately. As illustrated in melanoma, high pre-treatment Exo-PD-L1 is a biomarker for poorer response to immunotherapy, but a high on-treatment level of Exo-PD-L1 actually reflects a better response to immunotherapy [71]. Moreover, contradicting findings have complicated this issue. For instance, our study on NPC found that high on-treatment Exo-PD-L1 reflects a poorer response to M7824 [151], presenting a complete opposite from the result of the melanoma study [71]. Therefore, validation of the prognostic value of Exo-PD-L1 across more cancer types and in larger patient cohorts needs to be carried out, especially in NPC [153].

### 4.2. TEX in Predicting or Monitoring Chemo-Radiotherapeutic Response

In view of the impact of TEX cargos on therapeutic response, such as miR-106a-5p and circRNA Myc (CircMYC), studies have suggested the potential of quantifying specific miRNAs and proteins in serum exosomes as predictive markers of therapeutic response [154,155]. On the one hand, TEX may help to select patients who would respond to certain therapeutic agents; on the other hand, TEX can also serve the purpose of monitoring treatment effects because when cancer cells are exposed to acute stressors such as chemotherapy drugs and radiation, the function and composition of their exosome cargo will change accordingly [156]. This allows for TEX to reflect the changes in tumour cells over the course of treatment. As shown in head and neck squamous cell carcinoma (HNSCC), TEX, in response to radiation, increased the transmission of pro-survival and DNA repair factors through exosome cargos [157]. Also, in glioblastoma, TEX increased connective tissue growth factor (CTGF), which is involved in tumourigenesis, as observed post radiation exposure [158]. A study on prostate cancer also found that patients responding to neoadjuvant radiotherapy had significantly more miR-145 [159]. Similar findings were shown with miR-521 and miR-34c, which are miRNAs associated with TEX [160]. It is therefore increasingly pointed out that therapy-induced cellular changes can be reflected in exosomes. And such information can be exploited to monitor therapeutic response [13,161].

While NPC had been falling behind other cancers in exosome research, a recent study on NPC used TEX biomarkers to differentiate between radiosensitive and radioresistant patients [162]. Exosomes from different patient groups, i.e., responders and non-responders, have different spectral patterns. By recognising the unique spectral patterns of exosomes in liquid biopsy samples using surface-enhanced Raman spectroscopy (SERS), the researchers achieved an accuracy of 92.4% in stratifying the patients [162]. These findings suggest that exosome content can be a biomarker for identifying responders and monitoring treatment response.

### 4.3. Circulating TEX miRNA in Diagnosing NPC

NPC-TEX in the circulation harbour an altered profile of miRNAs due to functional changes in their originating NPC cells, which can be of diagnostic value. Jiang, L et al. identified three miRNAs (miR-134-5p, miR-205-5p, and miR-409-3p) with strong diagnostic potential for NPC patients [163]. The three-miRNA model showed an area under the ROC curve (AUC) of 0.91 in ROC analysis. It also retained good diagnostic performance on different NPC subgroups (EBV+, EBV−, early stage, and advanced stage). This demonstrates the potential of exosomal miRNAs as diagnostic biomarkers for NPC patients of different subtypes and stages [163]. Zheng et al. identified 21 upregulated and 10 downregulated miRNAs in patient plasma exosomes. miR-1301-3p was determined to be more associated with NPC progression among the differential expressed (DE) miRNAs. Thus, circulating exosomal miR-1301-3p could also potentially be a diagnostic biomarker for NPC [164]. Of note, the discordance of DE miRNAs may suggest the need for larger scale studies to reveal a universal panel for the development of a scalable diagnostic kit.

While many are looking into intrinsic host miRNAs, Ramayanti, O et al. focused on EBV-encoded miRNAs in the circulation for diagnosis [165]. BART13-3p showed high specificity for NPC detection, which performed better than other diagnostic methods, including ELISA-based EBV-EBNA1-IgA and EBV DNA-load measurements. Also, the authors suggested that a combination detection method of BART13-3p with cell-free EBV-DNA fragment can improve the screening sensitivity and specificity [165].

### 4.4. New Techniques for Exosome Detection Diagnostics

In addition to miRNAs, circulating TEX also carry specific proteins which can be utilised for diagnosis. However, peripheral blood usually contains a very low proportion of TEX, especially in early-stage tumour, due to the limited TEX-producing tumour cells. As a result, there is a low availability of specific TEX surface or enclosed proteins in whole blood [166]. Consequently, traditional detection methods such as ELISA and flow cytometry face difficulties in accurately detecting TEX. Therefore, there is a need for development of new methodologies to overcome these limitations.

A recent study has highlighted the utility of nanoflow cytometry (nFCM) for high-resolution analysis of individual EVs, including exosomes as small as 40 nm [167]. Using single-particle enumeration, they evaluated the concentrations of five exosome subsets characterised by two EBV-encoded latent membrane proteins (LMP-1 and LMP-2A) and three tumour markers (PD-L1, EGFR, and EpCAM). This approach achieved an accuracy of 96.3% in the testing cohort, surpassing the accuracy obtained by measuring EBV-VCA-IgA titre [167]. Furthermore, there are other studies featuring innovative Aptamer-based CRISPR/Cas12a and PLA-RPA-TMA assays. These assays utilise signal amplification techniques to improve the sensitivity of detecting exosomes with specific markers [168,169]. These studies have demonstrated promising results, with the Aptamer assay targeting a panel of CD109+ and EGFR+ exosomes achieving an AUC of 0.934, while the PLA-RPA-TMA assay targeting LMP-1 and EGFR achieved an AUC of 0.956 [168,169]. Interestingly, it was observed that the levels of these exosomes decreased in responders after radiotherapy. This again suggests that monitoring these TEX by their specific markers shows promise in therapeutic surveillance [168]. Moreover, even when comparing EBV-VAC-IgA-positive healthy people against early-stage NPC patients, the LMP-1/EGFR panel maintained an AUC of 0.906. This shows that exosome biomarkers could accurately differentiate early-stage NPC patients from not only completely healthy individuals, but also high-risk healthy individuals who tested positive for the EBV-VCA-IgA antibody [169]. Assays like the Aptamer can detect specific exosomes at a low concentration of 10^2 particles/mL, showing promise for early-stage cancer detection. However, the complexity and the potential costs of tailor-made testing reagents limit their practical use in population-wide screening. Additionally, it was also observed that NPC patients had a significantly higher concentration of total EVs compared to healthy donors. However, despite this difference, using total EV concentration as a diagnostic measure in nFCM achieved a sensitivity of only 64.3%, indicating that it may not be a reliable standalone diagnostic marker for NPC [167].

## 5. Limitations of Exosomes in Clinical Use and Future Directions

The evolving understanding of exosome-mediated pathogenic pathways within NPC TME provides inspiration for potential new treatment targets. Additionally, the novel techniques proposed to utilise exosomes as therapeutic agents and biomarkers also present promising opportunities. Nevertheless, the transition from bench to bedside faces multiple challenges. One primary obstacle is the extraction of exosome, which requires further refinement. Furthermore, there are still many aspects of NPC exosomes yet to be explored, highlighting the need for continued investigation in this field.

### 5.1. Difficulties in Extracting Exosomes

To fully unleash the potential of exosome-based liquid biopsies, it is crucial to develop cost-effective and efficient methods to isolate exosomes from patient body fluids like blood in high purity. Similarly, to obtain DEX/TEX for cancer vaccine, or to farm clean exosomes from mesenchymal stem cells for drug delivery, cost-effective methods to isolate exosomes from the source will be required. Currently, guidelines from the International Society for Extracellular Vesicles (ISEV) indicate that there is no single optimal method for separating exosomes [170]. The majority of published studies have used differential ultracentrifugation (UC) and size exclusion chromatography (SEC), which fall into the category of “intermediate recovery, intermediate specificity” according to ISEV. The term intermediate suggests that these methods have limitations such as inadequate yield and potential contamination from other free particles in the sample. Recent investigations have further suggested that in UC, exosomes are susceptible to damage caused by both shear force and manual handling during the multistep centrifugation process [171,172], whereas SEC is a time-consuming and complex method [173]. On the other hand, capture-based strategies offer advantages in terms of efficiency and purity. These approaches target specific proteins, such as tetraspanins (CD9, CD63, ALIX), and Ep-CAM on the surface of exosomes. By magnetic forces, they effectively capture the exosomes from samples with high levels of purity. The immunoaffinity capture (IAC-Exo) protocol, for instance, employs magnetic beads coated with antibody against Ep-CAM and demonstrated greater effectiveness compared to centrifuge-based methods [174]. However, it is important to note that capture-based techniques are limited to extracting specific populations of exosomes depending on the availability of suitable antibodies and the presence of specific exosome markers [174].

Nonetheless, these are all heavily laboratory based. To enable the transition to more general clinical usage, rapidly emerging microfluidics systems may be the silver bullet. These systems can incorporate various exosome isolation techniques while flowing the sample through narrow channels. The ExoSearch chip exemplifies the use of magnetic-based separation by injecting immunomagnetic beads into plasma samples that flow through the microfluidic device. With the help of an external magnetic field, the captured exosomes will be retained in a separate microchamber for further processing [175]. There are also capture-based chips where the inner wall of the microchannels is modified with anti-CD63 and anti-CD81 [176,177]. The advantage of microfluidic techniques is the possibility for extraction and analysis to be carried out on a single chip. For example, a method proposed by He et al. involves introducing a lysis buffer to the downstream of the exosome isolation step. After that, specific proteins from the lysed exosomes can be captured in a second chamber with another set of beads for analysis [178]. These microfluidic devices enable efficient extraction and detection of specific exosomes, making them suitable for use in clinical applications [179]. In relation to NPC, a successful separation of exosomes from the culture medium of C666-1 cell line using a microfluidic-based approach has been achieved by Teoh et al. [180]. However, it is important to note that the scalability of capture-based techniques is limited by cost constraints.

To broaden the clinical use of exosomes in NPC or other cancers, there is a need for improved isolation methods and an integration with exosome analysis tools in user-friendly devices like microfluidic chips. Acoustic-based separation, which separates particles of different sizes using varying acoustic forces, has shown promise as a novel method [181]. While simpler methods such as ultrafiltration also show potential, it is necessary to improve their yield [182].

### 5.2. Safety Concerns of Exosome-Based Therapy

To date, there are no more than 20 clinical trials using exosomes sourced from MSCs, DCs, and plants as treatment agents, with the majority of them still being in phase I [183]. Moreover, there are currently no FDA-approved exosome products. Therefore, the risks associated with exosome therapy are pretty much unknown. One possible concern might be contamination. As discussed, most of the exosome delivery systems being developed utilise MSCs as a source. Kalluri et al., for instance, developed a bioreactor to produce clinical-grade exosomes from MSCs in a large scale and listed a clinical trial of exosomes targeting Kirsten rat sarcoma viral (Kras) in pancreatic cancer [184]. However, if the exosomes in use are not correctly extracted and “sterilised”, they can contain genetic materials from the origin MSCs. These materials can potentially be harmful. Studies have found that MSC-derived exosomes can affect the activities of lymphocytes, macrophages, neutrophils, DCs, and NKs to reduce their secretion of pro-inflammatory cytokines [185,186]. In addition, MSC-derived exosomes can also promote the differentiation of CD4+ T cells into Th2 and Tregs, reducing Th1 and Th17 [187,188]. In the context of cancer treatment, such immunosuppressive effects could be detrimental. Furthermore, exosome surfaces are often modified such that they can specifically fuse with target cells. While there are successful cases of targeted delivery [189], exosomes may lose their specificity due to unexpected progresses like degradation of the surface peptide and result in side effects [190]. Particularly, if we were to use modified TEX in treatments such as cancer vaccines, the issue of purification and specificity would be even more concerning as TEX contain miRNAs and cargos that could cause cancer or proliferate the already existing cancer cells [191,192,193].

### 5.3. Inadequate Understanding of Exosome–Cellular Interactions

To better target the exosome-mediated pathogenic pathways in NPC and reverse processes like immune evasion, a comprehensive understanding of the complex interplay between exosomes, tumour cells, immune cells, and stromal cells in NPC is needed. In NPC, one area that deserves particular attention is the role of exosomes in tumour immunity, which is currently under-explored. While previous work on NPC has focused on the effects of TEX on T cells and macrophages, other cancers have expanded their interest to NK cells, DCs, MDSCs, or even B cells [27,28,29,32]. Since immune cells are interactive, it is important to perform network analysis and identify the central regulators for exosome-based immunotherapy to target the culprit of immunosuppression. Non-tumour-derived exosomes also play a significant role in tumour progression and immune evasion, which has also been overlooked in the field of NPC. As documented in other cancers, exosomes derived from CAFs, Tregs, TAMs, and more can contribute to tumour immune evasion [143,150]. Moreover, other processes such as tumour progression and metastasis can also be affected by non-tumour-derived exosomes [194,195,196]. Although there are some novel studies on the effects of exosomes derived from endothelial cells and platelets on metastasis and therapy resistance in NPC, such work is limited [197,198]. In future studies of NPC, it is important to diversify these directions and avoid an excessive focus solely on TEX.

## 6. Conclusions

Exosomes are nanoparticles which play vital roles in intercellular communications within the TME in a myriad of tumours, including NPC. Importantly, exosomes derived from host tumour, immune, or even other stromal cells can themselves be retrieved or biologically engineered and utilised as anti-tumour agents. Although the potential of exosomes as cytotoxic agents or drug delivery systems has been highlighted, their practical usage is still limited by challenges in effectively isolating them and addressing safety concerns through clinical trials. Likewise, the presence of TEX in biofluids of patients has shed light on the development of new, non-invasive biomarkers, but the protocol for exosome extraction and characterisation from complex fluid samples is yet to be standardised. Concerns regarding efficiency, purity, complexity, costs, and scalability will have to be addressed in the future to make the generalisation of exosome-based liquid biopsy possible. To summarise, exosomes hold significant value in improving our understanding of NPC and introducing new therapies or disease-monitoring tools to the clinical world, but their mobilisation into actual practice still has a long way to go.

## Figures and Tables

**Table 1:** TEX-mediated pathways leading to tumour progression and metastasis in NPC.

Pathogenic Mechanism	Exosome Cargo(s)	Target Gene(s)/Pathway(s)	Effects of Exosome Cargo(s) in Recipient Cells	Ref.
1a. Angiogenesis				
VEGF Upregulation in ECs	ICAM-1	MAPK	Mediates neovascularisation through Src kinase, ERK1/2, p38 MAPK, RhoA/ROCK and eNOS.	[80]
	miR-17-5p	BAMBI	miR-17-5p downregulate BAMBI, leading to disinhibition of Akt, and thus increased downstream VEGF-A expression.	[81]
	miR-144	FBXW7/HIF-1 α Axis	miR-144 upregulates VEGF-A via the FBXW7/HIF-1α axis.	[82]
Other Mechanisms to Alter ECs’ Properties	STIM1/LMP-1	Akt/ERK	EBV LMP-1 promotes proliferation, migration, tubulogenesis and permeability in ECs by activating the Akt/ERK pathway. STIM1 was found to promote LMP-1 enrichment in NPC-TEX.	[83]
	miR-23a	TSGA-10	miR-23a represses TSGA10 and positively regulates ERK signalling, resulting in proliferation, migration, and formation of tube-like structures in ECs.	[84]
	miR-205-5p	DSC2	miR-205-5p downregulates tumour suppressor DSC2 to promote EGFR/ERK signalling and enhance ECs’ proliferation.	[85]
	CCAT2	Unknown	The lncRNA boosts the proliferation and migration ability of ECs.	[86]
	lincROR	p-AKT, p-VEGFR2	Accelerate the growth of blood vessels, contributing to proliferation, migration and tube formation ability.	[87]
	PFKFB3	ERK, p-AKT	PFKFB3 improve vessel sprouting by regulating cytoskeleton remodelling, migration and tip cell competitiveness.	[88]
	HAX-1	ITGB6	By upregulating ITGB6, the FAK pathway is activated, leading to higher cell junction permeability and neovascularisation.	[89]
	miR-455	ZO-1	Under hypoxia, the exosomal miR-455 level is increased, which increases vascular permeability via the inhibition of ZO-1, a protein for endothelial tight junctions.	[90]
ECM Modulation	CD44v5	Adhesive Proteins	Mediates endothelial cell adhesive proteins and their interactions with ECM components.	[80]
	EBERs	VCAM-1	EBERs delivered into ECs are recognised by cytoplasmic TLR-3/RIG-I, activating the downstream ERK1/2/AP1 axis, thus stimulating VCAM-1 adhesive protein expression.	[91]
	miR-205-5p	DSC2	Inhibition of DSC2 by miR-205-5p enhances EGFR/ERK signalling and MMP-2, -9 expression such that extracellular matrix proteins are degraded and remodelled.	[85]
1b. Metastasis				
Promoting Tumour Intravasation	HMGA2	Snail	HMGA2 is overexpressed in EBV-infected NPC and their TEX. It upregulates Snail in ECs, promoting mesenchymal transition and degrading tight junctions, increasing vascular permeability.	[92]
Inducing EMT in NPC Cells	MMP-13	AKT1, ERK1/2	NPC-TEX is often enriched in MMP-13. Overexpression of HIF-1α in NPC cells under hypoxia is a probable cause. Exosomal MMP-13 could induce EMT in normoxic tumour cells possibly through AKT1 and ERK1/2 signalling.	[93]
	miR-106a-5p	FBXW7- TRIM24- SRGN Axis	Exosomal miR-106a 5p suppresses FBXW7 to downregulate FBXW7-mediated ubiquitin degradation of TRIM24. Thus, more TRIM24 binds to SRGN promoter region, and SRGN then promotes metastasis through EMT.	[94]
	miR-18a-5p	BTG3	EMT markers are induced by TEX miR-18a-5p in NPC cells by suppressing BTG3 and activating the Wnt/β-catenin pathway.	[95]
	miR-301a-3p	BTG1	Aberrant expression of miR-301a-3p promotes the proliferation, migration, invasion and EMT of NPC cells by suppressing BTG1 mRNA, a tumour suppressor gene.	[96]

Abbreviations: “AP1: activator protein 1; AKT1: RAC-alpha serine/threonine-protein kinase; Akt: serine-threonine kinase; BAMBI: BMP and activin membrane-bound inhibitor; BTG: B-cell translocation gene; CCAT2: colon cancer-associated transcript 2; CD44v5: CD44 variant 5; DSC2: desmocollin 2; EBERs: Epstein–Barr virus-encoded small RNAs; ECM: extracellular matrix; ECs: endothelial cells; EGFR: epidermal growth factor receptor; EMT: epithelial-to-mesenchymal transition; eNOS: endothelial nitric oxide synthase; ERK1/2: extracellular signal-regulated kinase 1/2; FBXW7: F-box/WD repeat-containing protein 7; HAX-1: HS1-associated protein X-1; HIF-1α: hypoxia-inducible factor 1 subunit alpha; HMGA2: high mobility group AT-hook 2; ICAM-1: intercellular adhesion molecule-1; ITGB6: integrin beta 6; LMP-1: latent membrane protein 1; lincROR: long intergenic non-protein coding RNA, regulator of reprogramming; MMP: matrix metalloproteinase; p38 MAPK: p38 mitogen-activated protein kinases; PFKFB3: 6-phosphofructo-2-kinase/fructose-2,6-bisphosphatase 3; ROCK: Rho-associated kinases; RIG-I: retinoic acid-inducible gene I; ROS: reactive oxygen species; SRGN: serglycin; STIM1: stromal interaction molecule 1; TLR-3: Toll-like receptor 3; TRIM24: Tripartite Motif Containing 24; TSGA-10: testis-specific gene antigen 10; TSP-1: thrombospondin 1; VCAM-1: vascular cell adhesion molecule 1; VEGF: vascular endothelial growth factor; ZO-1: zonula occludens-1”.
